# Oxidative stress-related risk of the multiple sclerosis development

**DOI:** 10.5937/jomb0-37546

**Published:** 2023-01-20

**Authors:** Marija Vasić, Aleksandra Topić, Bojan Marković, Neda Milinković, Evica Dinčić

**Affiliations:** 1 Military Medical Academy, Institute of Medical Biochemistry, Belgrade; 2 University of Belgrade, Faculty of Pharmacy, Department of Medical Biochemistry, Belgrade; 3 University of Belgrade, Faculty of Pharmacy, Pharmaceutical Chemistry, Belgrade; 4 Military Medical Academy, Neurology Clinic, Belgrade

**Keywords:** multiple sclerosis, oxidative stress, oxidative stress index, 8-oxo-7, 8-dihydro-2'-deoxyguanosine, total antioxidant status, total oxidative status, multipla skleroza, oksidativni stres, indeks oksidativnog stresa, 8-okso-7, 8-dihidro-2'-deoksiguanozin, ukupni antioksidativni status, ukupni oksidativni status

## Abstract

**Background:**

Multiple sclerosis (MS) is characterized by inflammation, demyelination and axonal degeneration. Oxidative stress (OS) plays a significant role in the pathogenesis of the disease. The aim of the study was to examine the association between OS and smoking on the MS development.

**Methods:**

The study included 175 patients with relapsing-remitting multiple sclerosis (RRMS) (76 males, 99 females) and 254 healthy subjects (81 males and 173 females). Oxidative stress biomarkers in serum, Total Antioxidant Status (TAS) and Total Oxidative Status (TOS) were determined spectrophotometrically. Oxidative Stress Index (OSI) was calculated as the ratio of TOS and TAS. Urinary 8-oxo7,8-dihydro-2'-deoxyguanosine were determined by HPLC-MS/MS and expressed as 8-oxodG/creatinine.

**Results:**

In females with RRMS were higher TOS, OSI and 8-oxodG/creatinine than in females in control group. The group of males with RRMS had lower level of TAS than the males in control group. Higher levels of 8-oxodG/creatinine was obtained in active, passive and former smokers with RRMS than in control group with the same exposition to tobacco smoke. Independent predictors of MS are passive smoking, increased OSI and increased levels of urinary 8-oxodG/creatinine.

**Conclusions:**

Our results demonstrate that the OS parameters should be included in the assessment of the risk for MS development. Due to the more sensitivity to oxidative stress, females may be at higher risk of MS development. This data indicates the importance of introducing the antioxidant therapy as a complementary treatment in patients with RRMS.

## Abstract (sr)

**Uvod:**

Multipla skleroza (MS) se karakteriše upalom, demijelinizacijom i degeneracijom aksona. Oksidativni stres (OS) igra značajnu ulogu u patogenezi bolesti. Cilj studije je bio da se ispita povezanost OS i pušenja na razvoj MS.

**Metode:**

Studija je obuhvatila 175 pacijenata sa relapsnoremitentnom multiplom sklerozom (RRMS) (76 muškaraca, 99 žena) i 254 zdrava ispitanika (81 muškarac i 173 žene). Biomarkeri oksidativnog stresa u serumu, ukupni antioksidativni status (TAS) i ukupni oksidativni status (TOS) su određeni spektrofotometrijski. Indeks oksidativnog stresa (OSI) je izračunat kao odnos TOS i TAS. Urinarni 8-okso7,8-dihidro-2'-deoksiguanozin je određen HPLC-MS/MS i izražen kao 8-oksoG/kreatinin.

**Rezultati:**

Kod žena sa RRMS bili su viši TOS, OSI i 8okodG/kreatinin nego kod žena u kontrolnoj grupi. Grupa muškaraca sa RRMS imala je niži nivo TAS od muškaraca u kontrolnoj grupi. Veći nivoi 8-okodG/kreatinina su dobijeni kod aktivnih, pasivnih i bivših pušača sa RRMS nego u kontrolnoj grupi sa istom izloženošću duvanskom dimu. Nezavisni prediktori MS su pasivno pušenje, povećan OSI i povećani nivoi 8-okodG/kreatinina u urinu.

**Zaključak:**

Naši rezultati pokazuju da parametre OS treba uključiti u procenu rizika za razvoj MS. Zbog veće osetljivosti na oksidativni stres, žene mogu biti izložene većem riziku od razvoja MS. Ovi podaci ukazuju na značaj uvođenja antioksidativne terapije kao komplementarnog lečenja kod pacijenata sa RRMS.

## Introduction

Multiple sclerosis (MS) is a chronic inflammatory, demyelinating, and neurodegenerative disease of the central nervous system. Epidemiological studies have shown the increasing prevalence of MS in every world region during last decade (2013.-2021.) [1]. Also, the prevalence of the disease shows gender dependence and female to male ratio is around 2: 1, with the average age of MS patients between 20 and 40 years [2]. There are several types of MS and the most common is relapsing-remitting multiple sclerosis (RRMS) (approximately 87%) followed by changing the phases of relapse and remission [3].

The cause of MS remains unknown, but the main etiological factor is inflammation. Also, the activation of microglia and macrophage cells initiating oxidative stress (OS), neurodegeneration and demyelination [4] [5]. OS occurs as a consequence of deficiency of antioxidant protection, and overproduction of free radicals as unstable, reactive molecules that leading to the oxidation of lipids, proteins, DNA molecules and cell damage [6] [7]. Research shows that smoking is associated with a risk of developing MS and progression at the population level. Also, studies found that cigarette smoking not only increases the susceptibility towards MS but may also contribute to rapid disease progression [8] [9]. Tobacco smoke contains free radicals, carbon-monoxide (CO), cyanides and other toxic chemicals which stimulate imflammatory cells to produce free oxygen radicals and consequently lead to demyelination and neurodegeneration [10]. Hedström et al. [11] noted a clear dose-response relationship between cumulative smoking dose and MS risk.

Biomarkers important in the assessment of the OS are Total Antioxidant Status (TAS), Total Oxidation Status (TOS) and Oxidative Stress Index (OSI) as the most reliable to assess the balance between circulating oxidants and antioxidants [12]. The raising evidence is that biomarker of oxidative DNA damage, 8-oxo-7,8-dihydro-2'-deoxyguanosine (8-oxodG) is potentially significant in MS pathology. 8-oxodG is biomarker of systemic effect of oxidative stress with the pro-mutagenic potential [13].

The aim of this study was to examine the effect of oxidative stress on the occurrence and develop-ment of multiple sclerosis in the population of the Serbia.

## Materials and methods

### Subjects

The study was conducted at the Clinic of Neurology, Military Medical Academy (MMA), Belgrade, Serbia, in the period from March 2018 to July 2019. Serum and urine samples were obtained from 175 patients with relapsing-remitting multiple sclerosis (RRMS) (76 males and 99 females, age range of 34-48 years). Of the total number of MS patients, 111 were treated with immunomodulatory therapy and 64 were without therapy. Criteria for exclusion of participants from the study were the presence of Primary Progressive MS (PPMS), diabetes mellitus, heart disease, and autoimmune disease. The control group included the 254 healthy subjects (81 males and 173 females, age range 28 ± 45). Criteria for exclusion of participants were the presence of neurological, inflammatory, and autoimmune chronic diseases.

All participants gave their informed consent to join the study. The study was performed in conformance with the Declaration of Helsinki ethical guidelines and the protocol was approved by the local Ethic Committee (Ethics Committee of the Military Medical Academy (MMA) of the MMA No.4494-1 from 01.04.2016).

### Biochemical analyses

Biomarkers of oxidative stress were measured in the serum and urine of MS patients and healthy subjects. The urine was used to determine biomarker 8-oxodG/creatinine. The serum was used to determine biomarkers TAS and TOS. Oxidative stress index (OSI) was calculated using the formula:

OSI (arbitrary unit) = [TOS (mmol H_2_O_2_ Eq/L)/<br>TAS (mmol Trolox Eq/L)] x 100 [14].

Blood samples were colected after overnightfasting, using a Beckton & Dickinson Vacutainer^®^Blood Collection Tubes. After spontaneous coagulation, blood samples were centrifugated at 850xg (3000 rpm) for 15 minutes and serum aliquots were stored at -80°C for later use. Urine samples were centrifugated at the 850xg (3000 rpm) for 15 minutes and stored at -80°C for later use. Before analysis, the samples were left at 3-8°C to thaw spontaneously. Then, they were homogenized on the vortex mixer and sonificated 2 minutes in an ultrasonic bath, centrifuged at 10000xg for 10 minutes and analyzed.

The urinary 8-oxodG was determined using high performance liquid chromatography and tandem mass spectrometry (HPLC-MS/MS) [15]. Solutions of ammonium acetate and acetonitrile were used as a mobile phase. Measurement of 8-oxodG in urine was performed on Thermo Accela (Thermo Scientific, Waltham, Massachusetts, USA), coupled to a triple quad Mass Spectrometer Thermo TSQ Quantum Access Max (Thermo Scientific, Waltham, Massachusetts, USA) with a heated electrospray ionization (HESI) interface. Urine samples were mixed, sonicated 2 minutes and then centrifugated for 10 minutes at 10000xg. The 20 μL of supernatants were injected in a thermostatted HPLC autosampler at 10°C. Results are expressed in a relation to the concentration of creatinine in first morning samples of urine as nmol/mmol. Analysis of the creatinine in urine was performed using a biochemical analyser Advia 1200 (Siemens Healthcare Diagnostic, Tarry town. NY, USA) by the Jaffe's kinetic method.

The level of TOS in the serum of patients and healthy subjects was determined using a modified Erel's spectrophotometric method [12] applied to the biochemical analyzer Olympus AU400^®^ (Beckman Coulter, Inc. USA). Oxidants from the sample oxidize ferrous ion-dianisidine complex to ferric ion in acidic medium. The oxidation reaction is facilitated by the presence of glycerol molecules in the reaction medium. The results are determined from the standard curve, and were expresses as mmol H_2_O_2_ Equiv/L.

The concentration of biomarker TAS was determined by modified Erel's spectrophotometric method [16]. The principle of the reaction is based on the oxidation of a stable chromogen, 2,2'-azinobis-(3-ethylbenzthiazoline)-6-sulfonic acid (ABTS), to the corresponding cation (ABTS˙^+^) using hydrogen peroxide in an acidic environment, which creates an emerald color. The ABTS˙^+^ is decolorized by antioxidants according to their concentrations, which is manifested as a decrease in color intensity at 660 nm. The intensity of discoloration is proportional to the concentration of total antioxidants in the sample. The method was applied to the biochemical analyzer Olympus AU400^®^ (Beckman Coulter, Inc. USA). The reaction rate is calibrated with Trolox (6-hydroxy-2,5,7,8-tetramethyl chroman-2-carboxylic acid, the water-soluble analogue of vitamin E), and results were expressed as mmol Trolox Equiv/L.

### Statistics

Statistical analysis was performed in SPSS Statistics for Windows, Version 20.0. (SPSS Inc. Chicago, IL. USA). The normality of data was assessed with Kolmogorov-Smirnov test. The date shows a normal distribution, and they are displayed as a mean ± standard deviation. In that case, parametric statistical tests of comparison of mean values between two groups (Student-t-test), as well as test of mean values between three groups (ANOVA, one factor, two factors ANOVA) were applied. The student's-t-test was used to examine the independent data of the two groups.

If the existence of a statistically significant difference was determined by the two-factor ANOVA test, the Post hoc test according to Sidak was used in the further analysis. A logistic regression analysis was employed to test possible predictors of MS. The significance level for all statistical tests was set at p<0.05.

## Results

The demographic characteristics, smoking status, and oxidative stress parameters in the control group and MS patients, as well as according to the sex, was presented in Table 1.

**Table 1:** Demographic characteristics and parameters of oxidative stress in all healthy subjects and MS patients and according to sex. Quantitative variables are presented as mean ± Sd; ^*^ age of patients when was diagnosed the MS; TOS: total oxidative status; TAS: totalantioxidant status; OSI: oxidative stress index; 8-oxodG: 8-oxo-7,8-dihidro-2-deoxiguanosine; ^a^ differences between all participants in control group and MS group; ^b^ differences between control and MS in male population; ^c^ differences between control and MS in the group in female population; ^d^ differences between males and females in the MS group; ^e^ differences between males and females in the control group.

Biomarker	All		Males		Females	
	Control group<br>(n=254)	MS patients<br>(n=175)	Control group<br>(n=81)	MS patients<br>(n=76)	Control group<br>(n=173)	MS patients<br>(n=99)
Age (years)^*^	36.90±8.78^a^	33.43±9.10	37.41±7.82^b^	33.22±8.05	36.62±9.21^c^	33.60±9.93
MS duration (years)	-	7.56±5.60	-	7.81±4.72	-	7.41±8.72
Males, n (%)<br>Females, n (%)	81 (31.9)^a^<br>173 (68.1)	76 (43.4)<br>99(56.6)	-	-	-	-
Non-smokers, n (%)<br>Former smokers, n (%)<br>Passive smokers, n (%)<br>Current Smokers, n (%)	140 (55.1)<br>24 (9.4)^a^<br>6 (2.4)^a^<br>84 (33.1)	77 (44.0)<br>4 (2.2)<br>40 (22.8)<br>56 (32.0)	48 (59.2)<br>6 (7.4)<br>2 (2.5)^b^<br>25 (30.9)	34 (44.8)<br>1 (1.3)<br>16 (21.0)<br>25 (32.9)	92 (53.2)<br>18 (10.4)^c^<br>4 (2.3)^a^<br>59 (34.1)	43 (43.4)<br>3 (3.1)<br>24 (24.2)<br>29 (29.3)
TAS<br>(mmol Trolox Equiv/L)	2.02±0.21	2.00±0.20	2.18±0.18^b,e^	2.09±0.21^d^	1.94±0.18	1.93±0.17
TOS<br>(μmol H_2_O_2_ Equiv/L)	8.67±1.85^a^	9.02±1.76	8.38±1.99	8.55±1.67^d^	8.81±1.76^c^	9.40±1.76
OSI<br>(arbitrary units)	0.43±0.10^a^	0.45±0.10	0.39±0.10^e^	0.41±0.09^d^	0.45±0.09^c^	0.49±0.10
8-oxodG/ creatinine<br>(nmol/mmol)	1.18±0.56^a^	1.58±1.09	1.31±0.49^e^	1.44±0.72	1.12±0.58^c^	1.70±1.31

There were significantly more males in the group of MS patients compared to the control group (43% and 32%, respectively; p=0.015). Patients were younger when diagnosed with MS compared to healthy participants (33-and 37-year-old, respectively; p<0.001). The same differences were obtained in males (33-and 37-year-old, respectively; p=0.001) and females (34 and 37-year-old, respectively; p=0.016).

Passive smoking was significantly more common in patients than in control group (23% and 2.4%, respectively; p<0.001; odds ratio, 95% CI: 5.06> 12.25>29.62), which was also obtained in the male population (21% and 2.5%, respectively; p=0.0002; odds ratio, 95% CI: 2.33>10.53>47.58), and females (24% and 2.3%, respectively; p<0.001; odds ratio, 95% CI: 4.53>13.52>40.33). This data show that passive smoking is associated with a 10 times higher risk of developing MS in males and 13 times higher risk in females than in healthy males and females. In the group of MS patients there was a significantly lower frequency of former smokers than in control group (2.2%, and 9.4%, respectively; p=0.003; odds ratio, 95% CI: 0.08>0.22>0.65), which was also obtained in the female population (3.1%, and 10.4%, respectively; p <0.001; odds ratio, 95% CI: 0.09>0.29>0.96).

In the group of the all-MS patients were higher levels of pro-oxidative biomarkers in comparison to control group (TOS, p=0.018; OSI, p=0.023; 8-oxodG/creatinine, p<0.001). The significantly increased pro-oxidative biomarkers in MS patients were obtained only in female population (TOS, p=0.010; OSI, p=0.006; 8-oxodG/creatinine, p<0.001), but not in males (TOS, p=0.571; OSI, p=0.137; 8-oxodG/creatinine, p=0.255). The group of males with MS had significantly lower level of TAS than the healthy males in control group (p=0.006). The gender-related differences in OSparameters were obtained in both MS patients, and in control group. The MS-females had lower level of TAS (p<0.001), higher levels of TOS (p=0.002) and OSI (p<0.001) than MS-males. In control group, females had lower level of TAS (p<0.001) and 8-oxodG/creatinine (p=0.030) and higher OSI (p<0.001), than males.

**Figure 1:**
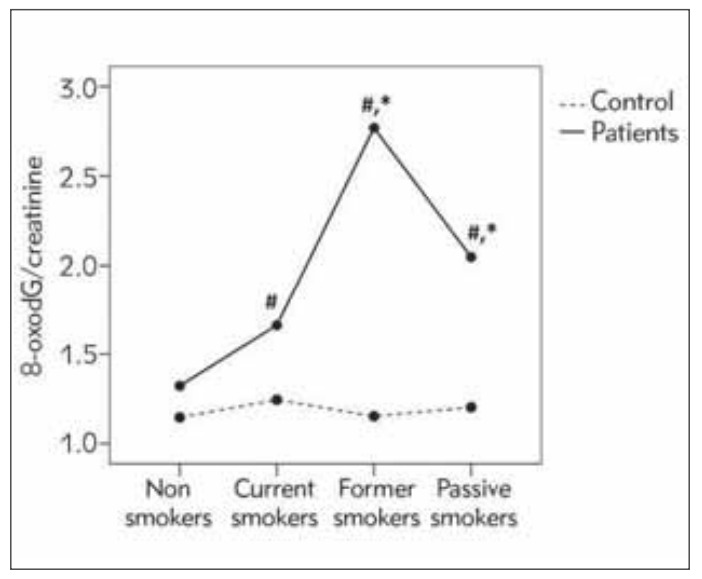
Estimated marginal means of 8-oxodG/creatinine(nmol/mmol); ^#^differences between control group and group of MS patients; ^*^MS group: difference in relation to the group of non-smokers

A two-way between-groups analysis of variance and Sidak adjustment post-hoc test, was conducted to explore the impact of smoking status (non smokers, former smokers, passive smokers, current smokers) and present of MS on the levels of 8-oxodG/creatinine. The results were presented in Figure 1. The interaction effect between smoking status and presence of MS on the level of 8-oxodG/creatinine was statistically significant (p=0.025; Partial Eta Squared 0.030). Sidak's post hoc test revealed that former smoking, passive smoking and current smoking were associated with higher levels of 8-oxodG/creatinine in MS group than in control group (former smokers, p=0.002; passive smokers, p=0.050; current smokers, p=0.012). Additionally, in group of MS patients, former smokers and passive smokers had higher levels of 8-oxodG/creatinine than in non-smokers (formersmokers, p=0.017; passive smokers, p<0.001).

Binary logistic regression analysis used for the identification of the independent MS risk predictors. The overall regression model was statistically significant χ^2^ (df 5; N_310_) = 54.37; p<0.001) (Table 2). This model of binary logistic regression is explained in between 16% (Cox and Snell R square) and 22% (Nagelkerke R squared) variance in the development of MS and correctly classified 69% of MS patients. The passive smoking, increased OSI index and increased 8-oxodG/creatinine are the independent predictors of MS development, as shown in Table 2. The model shown that the strongest predictors of MS onset were the increased OSI index (OR 20.12), and exposure to passive smoking (OR 10.83).

**Table 2:** Binary logistic regression model for predicting the occurrence of multiple sclerosis. β (S.E): regression coefficient b (standard error for β); OR (95% CI): odds ratio (confidence interval); statistical significance for p<0.05; OSI: oxidative stress index; 8-oxodG: 8-oxo-7,8-dihidro-2’-deoxiguanosine; χ^2^: chi-squared test.

Predictors	β (S.E.)	p	OR (95% CI)
Passive smoking	2.38 (0.57)	<0.001	10.83 (3.54–33.12)
OSI	3.00 (1.23)	0.014	20.12 (1.82–222.62)
8-oxodG/creatinine	0.69 (0.19)	<0.001	1.99 (1.35–2.92)
Constant	-2.86 (0.63)		
The whole model	χ^2^(df 5. N351) = 54.37; p < 0.001		

## Discussion

In a very recent review was suggested that the oxidative/nitroxidative stress as an important risk factor is involved in the pathophysiology of multiple sclerosis [4]. Furthermore, the oxidative/nitroxidative stress is responsible for the MS progression and clinically presented symptoms. The data suggest that the oxidative damage of oligodendrocytes and neurons is associated with inflammation, and play a major role in neurodegeneration [17] [18]. Despite the numerous studies, the pathogenesis of the MS has not yet been completely elucidated. We aimed to examine the role of the OS in the occurrence of MS in the population of Serbia, given that the trend of increasing prevalence of MS in the population of Serbia was found [19].

We obtained that the all patients with MS had higher levels of the pro-oxidative biomarkers, TOS, OSI and 8-oxodG/creatinine, compared to the control group. The same differences were found only in female group, but not in males. A possible reason for this gender difference is the greater sensitivity of females for development of the MS caused by the oxidative stress. However, in males with MS the TAS level was significantly lower than in the control group. Although the males with MS had a reduced level of antioxidant protection than the healthy males, there was no development of OS. The studies by Kirbas et al. [20] and Acar et al. [21] also found depletion of anti-oxidative protection (decreased levels of TAS) and the higher levels of TOS and OSI in MS patients compared to the control group, regardless the gender. The gender-related difference in OS parameters obtained in our study is probably due to the larger number of samples than in the previously mentioned studies. Moreover, our study was confirmed that the increased OSI, and increased levels of 8-oxodG/creatinine are independent predictors of the MS development. This result supports the fact that the development of MS is associated with oxidative stress, caused by the disturbed the balance between oxidants and antioxidant protection.

It was interesting that the gender-related differences in OS were found independently in both control and MS patients. Our data shown that healthy females had the higher oxidative stress estimated by the OSI index than males, while in the healthy males was presented higher urinary 8-oxodG/creatinine than in healthy females. Elevation of the levels of 8-oxodG/creatinine may be an indicator of a long-term exposure of ROS which leads to DNA damage, and consequently to OS-related diseases [22] [23]. Examining the effect of smoking in healthy subjects, Mesaros et al. [24] found increased levels of 8-oxodG in smokers and suggested it as a useful biomarker in assessing oxidative stress caused by smoking. In group of MS patients, we noticed that females had lower TAS and higher levels of TOS and OSI than males. These results indicate the importance of the prevention of the oxidative stress in both sexes in order to prevent the occurrence of MS. These data indicates the necessity of antioxidative therapies in MS patients, especially for females. Our data contribute to the attitude that antioxidant therapy as adjunctive therapy in multiple sclerosis could contribute to the reduction or even prevention of the progression of MS, since the oxidative damage is involved in inflammatory and autoimmune-mediated processes [25]. In addition, the long-term negative effects of oxidative stress on oxidative modification of nucleic acids and the formation of pro-oncogenic DNA lesions, such as 8-oxodG, should be prevented. It has been shown that females are more susceptible to MS onset than males [26]. However, males are more likely to develop a severe form of the MS [27] and gray matter atrophy of the brain was higher in males than in females with MS [28].

We obtained more passive smokers and less a former smokers in the group of MS patients than in group of healthy subjects. Additionally, we found a significant increase in 8-oxodG/creatinine in MS patients who were active, former and passive smokers than in healthy subjects of the same smoking status. In addition, MS patients who were former and passive smokers had higher level of 8-oxodG/creatinine than MS patients who were non-smokers. This indicates more serious systemic effects of OS on DNA caused by the harmful prooxidans from tobacco smoke and the presence of the disease itself.

Literature data show that the smoking can play a significant role in the development of MS. Hedström et al. [29], states that smoking more than 10 packs od cigarettes a year, as well as long term exposure to tobacco smoke cause the three times risk of developing MS compared to non-smokers. Increased risk for the oxidative stress development caused by decrease of the plasma antioxidants in smokers have been documented by numerous studies [30] [31]. Sundström et al. [32] have shown that elevated cotinine (nicotine metabolite) was associated with an increased risk for MS predominantly in females. Additionaly, they found that modestly elevated cotinine levels suggestive of passive smoking are associated with an increased risk for MS, which was also confirmed in our study. According to them, smoke exposure may explain the higher incidence of MS in females. Our findings that passive smoking is associated with 10-fold higher risk of developing MS in males and 13-fold higher risk in females are in agreement with that study. Supporting of the fact that females are more susceptible to the MS onset, the study by Hewagama et al. [33] shown the differential expression of inflammatory/cytotoxic effector molecules in restimulated female T cells that may contribute to the differences in autoimmune diseases between females and males.

## Conclusion

The main result of the study in population of Serbia is that oxidative stress is associated with the risk of relapsing-remitting multiple sclerosis onset. Also, we showed that the females are in higher risk of RRMS onset because their great sensitivity to oxidative stress. This data indicates the importance of introducing the antioxidant therapy as a complementary treatment in patients with RRMS. Particularly sensitive are individuals who are exposed to tobacco smoke, regardless they are active, former or passive smokers, since they have elevated levels 8-oxodG/creatinine. Our study highlighted the importance of smoking cessation and reducing exposure to tobacco smoke in order to prevent development of MS. Also was emphasizes the importance of introducing laboratory biomarkers TOS, TAS, OSI and 8-oxodG/creatinine in order to assess the risk of developing of OS as the risk factor for MS development.

## Dodatak

### Author contributions

MV: analyzed the samples, analyzed and interpreted the data, and wrote the manuscript.<br>AT: made the design of the study, interpreted the data, made a critical review of the manuscript.<br>BM: analyzed the samples, interpreted the data.<br>NM: analyzed the samples, interpreted the data.<br>ED: made a critical review of the manuscript.

### Funding

This research was funded by the Ministry of Education, Science and Technological Development, Republic of Serbia through Grant Agreement with University of Belgrade-Faculty of Pharmacy No: 451-03-68/2022-14/200161.

### Ethical approval

The local Ethic Committee ofthe Military Medical Academy approved this study (No.4494-1 from 01.04.2016) and participants gave written informed consent.

### Acknowledgements

We would like to thank the nurses, and other healthcare professionals from the Neurology Clinic, Military Medical Academy, Belgrade, Serbia involved with data acquisition. Also, we thank the technicians for the help during the biochemical analysis from the Department of Medical Biochemistry University of Belgrade-Faculty of Pharmacy.

### Conflict of interest statement

All the authors declare that they have no conflictof interest in this work.
